# Apoptotic Lymphocytes of *H. sapiens* Lose Nucleosomes in GC-Rich Promoters

**DOI:** 10.1371/journal.pcbi.1003760

**Published:** 2014-07-31

**Authors:** Sergey Hosid, Ilya Ioshikhes

**Affiliations:** 1 Department of Biochemistry, Microbiology and Immunology, University of Ottawa, Ottawa, Ontario, Canada; 2 Ottawa Institute of Systems Biology, University of Ottawa, Ottawa, Ontario, Canada; Rutgers University, United States of America

## Abstract

We analyzed two sets of human CD4^+^ nucleosomal DNA directly sequenced by Illumina (Solexa) high throughput sequencing method. The first set has ∼40 M sequences and was produced from the normal CD4+ T lymphocytes by micrococcal nuclease. The second set has ∼44 M sequences and was obtained from peripheral blood lymphocytes by apoptotic nucleases. The different nucleosome sets showed similar dinucleotide positioning AA/TT, GG/CC, and RR/YY (R is purine, Y - pyrimidine) patterns with periods of 10–10.4 bp. Peaks of GG/CC and AA/TT patterns were shifted by 5 bp from each other. Two types of promoters in *H. sapiens*: AT and GC-rich were identified. AT-rich promoters in apoptotic cell had +1 nucleosome shifts 50–60 bp downstream from those in normal lymphocytes. GC-rich promoters in apoptotic cells lost 80% of nucleosomes around transcription start sites as well as in total DNA. Nucleosome positioning was predicted by combination of {AA, TT}, {GG, CC}, {WW, SS} and {RR, YY} patterns. In our study we found that the combinations of {AA, TT} and {GG, CC} provide the best results and successfully mapped 33% of nucleosomes 147 bp long with precision ±15 bp (only 31/147 or 21% is expected).

## Author Summary

We analyzed nucleosomal DNA of human CD4+ T normal and apoptotic lymphocytes. Dinucleotide positions (pattern) of AA/TT, GG/CC, WW/SS (W is adenine or thymine, S is guanine or cytosine) and RR/YY (R is purine, Y - pyrimidine) of nucleosome sequences in both cell conditions are similar and have period 10–10.4 bp. We successfully mapped 33% of nucleosomes with precision ±15 bp by combination of {AA, TT}, {GG, CC}, {WW, SS} and {RR, YY} patterns. We identified two types of promoters in *H. sapience*: AT and GC-rich. AT-rich promoters keep nucleosomes around transcription start site when GC-rich promoters lost 80% of nucleosomes during apoptosis at the same region.

## Introduction

A nucleosome is the basic unit responsible for packing eukaryotic DNA into chromatin. Nucleosome position is determined during chromatin assembly and related to regulatory elements of gene expression such as promoters, transcription factors and others. The precise position of a nucleosome is important for correct interaction and function of numerous gene regulatory elements. A nucleosome consists of a segment of DNA (∼146 bp) wound around a histone octamer protein core. One of the main factors that determine nucleosome position is a nucleosome DNA sequence pattern or variety of patterns. Specific patterns reflect DNA affinity to histone octamers and places nucleosomes at an essential position. Several models of nucleosome positioning have been published. The static model (model of DNA signal) describes the nucleosome affinity to specific periodical DNA patterns. Trifonov *et al.* [1] first obtained the 10.2 bp periodicity of certain dinucleotides in nucleosome DNA sequence. Specific patterns and/or periodicity were found in a variety of eukaryotic organisms such as *Saccharomyces cerevisiae* [2]–[6], *Caenorhabditis elegans* [7], *Homo sapiens* and D*rosophila melanogaster* [8], [9]. In addition to the periodicity, G+C content and the frequency of some AT-rich tetra-nucleotides influence nucleosome occupancy in vitro and in vivo in a manner comparable to Kaplan's model [5]. G+C content is dominant, alone explaining ∼50% of the variation in nucleosome occupancy in vitro [10].

Apoptotic DNA fragmentation is a key feature of apoptosis, a type of programmed cell death. Apoptosis is characterized by activation of endogenous endonucleases with subsequent cleavage of chromatin DNA into nucleosomal fragments of roughly 180 base pairs (bp) and multiples thereof (360, 540 etc.). The enzyme responsible for apoptotic DNA fragmentation is the Caspase-Activated DNase (CAD). CAD cleaves the DNA at the internucleosomal linker sites between the nucleosomes, protein-containing structures that occur in chromatin at ∼180 bp intervals [11]. Micrococcal nuclease (MNase) also can be used for cleavage of chromatin DNA to fragments of ∼150 bp, and has been often used for nucleosome mapping. Both enzymes display sequence specificity in their preferred cleavage sites, nucleosomes produced by CAD nuclease are 8–10 bp longer than those produced with MNase. The CAD cleavage sites tend to be 4–5 bp further away from the nucleosomal dyad than MNase cleavage sites [12].

We suggest that degradation of DNA in apoptotic cells is not a random process but is controlled by a specific chromatin related system. For this reason we have investigated and compared chromatin structure and nucleosome distribution in normal and apoptotic T-cells.

## Results

### 1. Distribution of nucleosomes around TSS, normal and apoptotic T-cells

**Figure 1:**
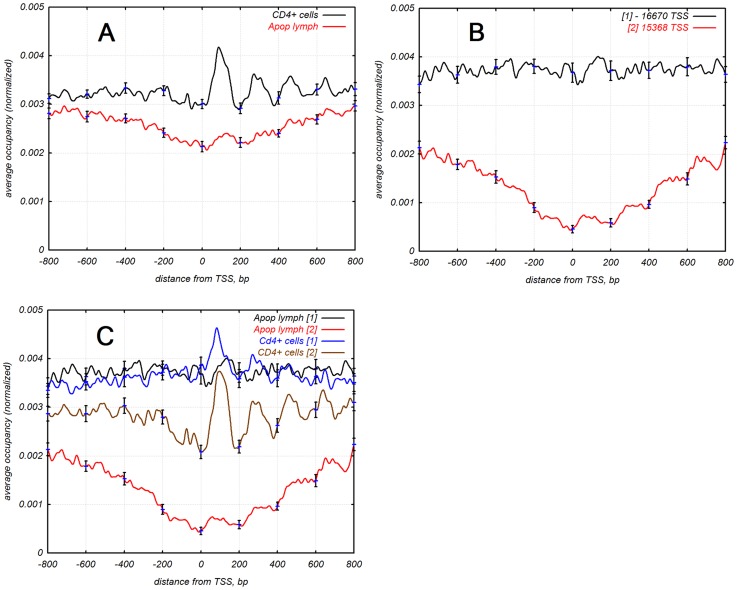
(**A**) **Distribution of nucleosomes around transcription start sites; blue line corresponds to apoptotic lymphocytes [14]; black line corresponds to normal CD4^+^ cells [8].** (**B**) Distribution of nucleosomes around transcription start sites; 32,038 promoters were divided into two groups by nucleosome occupancy in apoptotic lymphocytes [14], K-means clustering method: blue line corresponds to the first group of 16670 promoters with normal occurrence (one nucleosome per 250 bp) of nucleosomes; black line corresponds to the second group of 15368 promoters with low occurrence (one nucleosome per 1000 bp) of nucleosomes around TSS. (**C**) Distribution of nucleosomes around transcription start sites. Blue and red lines correspond to promoters of the first group with normal occupancy of nucleosomes, apoptotic lymphocytes and CD4^+^ cells respectively. These promoters have the similar average occupancy of nucleosomes, position of +1 nucleosome from set of apoptotic lymphocytes [14] is shifted 50–60 bp downstream. Black and orange lines correspond to promoters with low occupancy of nucleosomes during apoptosis, apoptotic lymphocytes and normal CD4+ cells respectively. These promoters lost 80% of nucleosomes around TSS during apoptosis.

Previous analysis of nucleosomal position around TSS in human showed that nucleosomes were well phased around TSS [8]. We analyzed nucleosomal distribution in the normal and apoptotic T-cells to figure out the stability and phasing of nucleosomes during lysis. Two sets of DNA nucleosomal sequences were matched on 32,038 human promoters (Fig. 1A). A well phased +1 nucleosome is observed in both data sets but its occurrence is low around TSS in apoptotic cells. We also performed the same analysis of four sets of human CD4+ cells obtained from [13].

### 2. Promoters with normal and low density of nucleosomes in apoptotic cells

We divided 32,038 promoters into two groups by distribution of nucleosome occupancy around TSS in apoptotic cells, using a K-means clustering method. Two different groups were clearly observed: the first group had the same distribution of nucleosomes around TSS as in normal CD4^+^ cells [8] while the second group had very low occupancy of nucleosomes around TSS (Fig. 1B).

The first group of promoters had a similar occupancy of nucleosomes in both normal CD4^+^ cells and apoptotic lymphocytes, where a +1 nucleosome (the first nucleosome after TSS) was phased as well. In apoptotic cells the position of +1 nucleosome was shifted 50–60 bp downstream compared to normal cells. The second group of promoters had a significant difference between normal CD4^+^ and apoptotic cells: promoters from the second group of apoptotic cells had less than 20% of nucleosomes around TSS than in normal CD4^+^ cell cells, Z-score = −4.4, P<0.001 (Fig. 1C).

Further analysis of each group revealed that the two groups had a different AT/GC ratio. The first group of promoters was AT-rich whereas the second was GC-rich (Supplementary Fig. S1a and S1b respectively).

Data of nucleosome occupancy in AT-rich (group 1) promoters in CD4+cells obtained by [13] have the similar distribution to the data obtained by [8]. With that nucleosome occupancy in GC-rich (group 2) promoters obtained from [13] has intermediate value between nucleosome occupancy in normal CD4+ cells obtained from [8] and nucleosome occupancy in the apoptotic lymphocytes obtained from [14]. (Supplementary Fig. S2).

A genome-wide analysis of nucleosome positioning in human apoptotic lymphocytes revealed the same phenomenon: AT-rich DNA retained nucleosomes whereas GC-rich DNA lost 80%, Z-score = −4.4 (P<0.001) of nucleosomes during apoptosis (Supplementary Fig. S3). Statistical significance was estimated by bootstrap method (see Methods).

### 3. Similarities and differences of dinucleotide nucleosomal patterns of two sets

**Figure 2:**
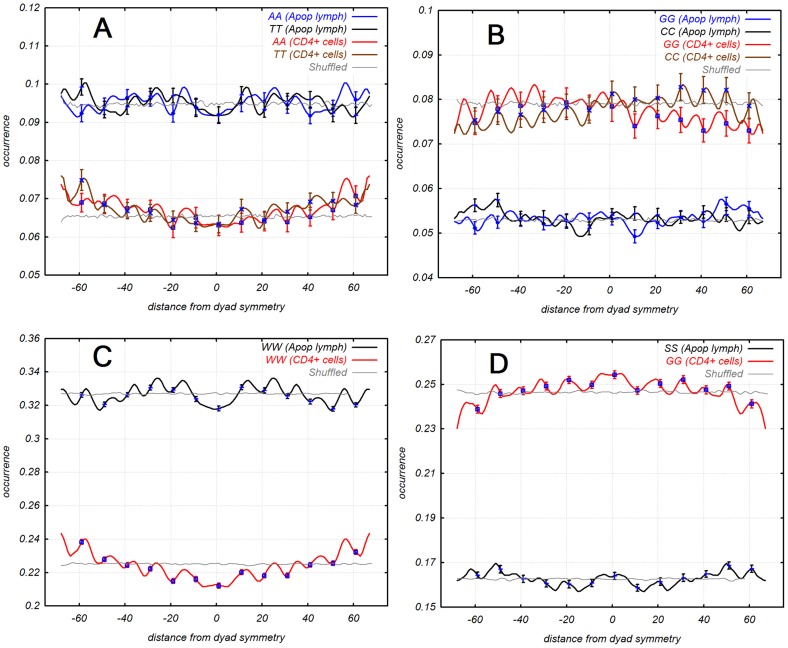
Dinucleotide distributions of (A) AA-TT and (B) GG-CC dinucleotides, (C) WW (adenine or thymine), and (D) SS (guanine or cytosine) around nucleosome dyad symmetry, whole sets. Apoptotic lymphocytes, data is from [14]; CD4^+^ cells, data is from [8].

A periodical distribution of ∼10 bp of AA-TT, GG-CC, WW-SS (A, T and G, C) and RR-YY (R is Purine, Y is Pyrimidine) dinucleotides were present in both sets (Fig. 2 and Supplementary Fig. S4, Fourier transformation). This observation was consistent with previous publications [15], [16]. The positions of the individual dinucleotide peaks were the same on the patterns of both sets. However, there were two significant differences between the dinucleotide distributions of the two sets. The first difference was that nucleosomal DNA sequences of apoptotic cells were more AT-rich than those of normal cells. This difference could be explained by the loss of nucleosome from GC-rich DNA during apoptosis. The second disparity was a convex curved gradient in the patterns of the normal CD4^+^ cells. GG, CC and SS (guanine or cytosine) dinucleotide patterns had maximum values at the symmetry dyad, whereas AA, TT and WW (adenine or thymine) dinucleotides had minimum values in this area. Purine-pyrimidine patterns of the two sets also had similarities: both have periodicity of ∼10 bp but did not have a convex curved gradient as WW-SS dinucleotides in normal cells (Supplementary Fig. S5).

We performed analysis of order and distances between dinucleotides of nucleosome patterns obtained from normal CD4^+^ cells by correlation between AA-TT; GG-CC; WW-SS (week and strong), and YY-RR (purine and pyrimidine) dinucleotide patterns (Supplementary Fig. S6). Correlation between all dinucleotides had a periodicity of 10–11 bp long. This may be because the short periodical nucleotide sub patterns 10–11 bp long form conventional nucleosome patterns 146 bp long. Alternation of purine and pyrimidine and guanine and cytosine has 5 bp and was consistent with previous publication [16], [17]. Adenine and thymine tended to form trinucleotide TTA, ATT, TAA, and AAT rather than tetranucleotide sequences AATT or TTAA. Alternation of AA and TT dinucleotide was 10 bp. This alternation and presence not only AAT and ATT but TAA and TTA trinucleotide was different from result obtained in [16] where observed patterns contained only T_n_A_n_ motif but not A_n_T_n_ motif. Guanine and cytosine tended to form only GGC and GCC trinucleotides but neither CGG or CGG trinucleotides, nor GGCC or CCGG tetranucleotides.

### 4. Pattern and position of +1 nucleosome

**Figure 3:**
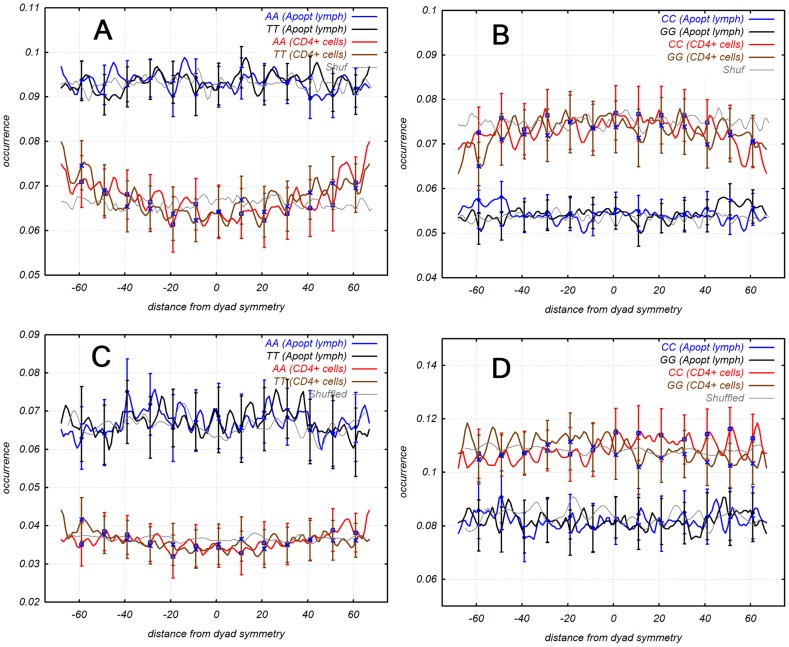
Dinucleotide distribution of +1 nucleosome. Group 1 (top panels) of apoptotic [14] + 1 nucleosomes has higher AT/GC ratio than set of normal CD4^+^ cells [8]. Set of CD4^+^ has phased AA, TT dinucleotides and AA-TT peak at the center of nucleosome. Group 2 is at the bottom panel. +1 nucleosome of CD4+ cells has counter phased GG and CC dinucleotide.

Nucleosomes were phased around TSS, especially the first (+1) nucleosome downstream from TSS. Position of nucleosomes can be effected by various factors such as affinity of histone octamer to specific DNA patterns and other factors related to transcription for example transcription factor binding proteins or chromatin structure remodelling complex [18], [19]. +1 nucleosome is adjacent to TSS and is involved in the process of gene regulation, so we anticipated that +1 nucleosome had different dinucleotide pattern than regular nucleosomes. We extracted a set of +1 nucleosome sequences (distance 90±50 bp downstream of TSS) from both cell types to test our hypothesis. Dinucleotide patterns of +1 nucleosome differed from other nucleosome patterns and varied between normal and apoptotic cells and between AT and GC-rich promoters as well (Fig. 3).

### 5. One or two linkers designate position of dyad symmetry

Two sets of nucleosome sequences, the first from [8] and the second from [14] were obtained using different methods. They had different length and number of flanking sequences of each DNA fragment. The set obtained by [8] contained short non paired (only one flank) sequences 24–25 bp long. The set obtained by [14] had paired (two flanks) sequences 120 bp long. There was a need to find the precise position of dyad symmetry of each set to obtain nucleosome patterns.

To determine the position of the dyad symmetry of the non paired ends set we calculate the correlation between patterns obtained from two strands of DNA 146 bp long. The position with maximum correlation corresponded to the position of the nucleosome. The set obtained by [8] had dyad symmetry at 75 bp from beginning of flank sequence; the set obtained by [14] had dyad symmetry at the 85 bp. This result is consistent with previous publications [12]. The average length of DNA fragments obtained from [8] was 150 bp long and 170 bp in the set obtained from [14] (Supplementary Fig. S7).

With two flanked sequences as in the set from [14] we can obtain the position of dyad symmetry using two methods: first by calculating maximum correlation between patterns of two DNA strands or by determining the midpoint between the two paired flanks. Analysis of fragments with different lengths revealed that the positions of dinucleotide patterns' peaks differ. Positions of the peaks were shifted correspondingly to the change in length of the fragments. For example if the length of a fragment increased by 10 bp the peaks shifted 5 bp to the left on the left side and 5 bp to the right on the right side (Supplementary Fig. S8). We suggest that nucleosomal patterns are dependent on the end of a fragment rather than on the midpoint between two ends. For this reason we aligned paired and non paired nucleosome DNA sequences by the 5′-ends of fragments from experimental data to obtain nucleosome patterns.

### 6. A typical nucleosome sequence has either AA-TT or GG-CC patterns

We matched two sets of nucleosome and one set of promoter sequences with the whole human genome and separated nucleosome sequences 313 bp long (including flank regions) from 32,038 promoter regions (±1000 bp from TSS), obtaining 711,873 nucleosomes from apoptotic and 581,507 nucleosomes from normal CD4^+^ cells. We obtained two 147 bp long 16-dinucleotides patterns from each set of nucleosome sequences (see Methods) to predict nucleosome positions around TSS. These two sets were mapped by the patterns {AA, TT, AT}, {GG, CC, GC}, {WW-SS}, and {RR, YY} (two last patterns are not shown). Patterns {AA, TT, AT} and {GG, CC, GC} detected 33% of nucleosomes with precision ±15 bp, {WW}, {SS} 29% and {RR-YY} 18% respectively. Combination of patterns {AA, TT, AT} and {GG, CC, GC} had the best result and could predict around 50% of experimental nucleosomes. Only 5% of nucleosomes were predicted by both {AA, TT, AT} and {GG, CC, GC} patterns. The maximum number of predicted nucleosomes matched with the center of nucleosome sequence, 60% more than with shuffled sequence, Z score = 10 (Supplementary Fig. S9).

We matched the set of nucleosome sequences from human CD4^+^ cells [8] with the whole human genome and separated 183,372 sequences 313 bp long (including flank regions) of the most precise nucleosomes (StDev = 0). This set was mapped by two {AA, TT} and {GG, CC} patterns obtained from normal human CD4^+^ cells and two {AA, TT} dinucleotide patterns of yeast nucleosomes obtained by [4] (Supplementary Fig. S10a–b). {AA, TT} and {GG, CC} patterns of normal human CD4^+^ cells predicted 50% and 48% of nucleosomes respectively in the interval ±36 bp (one fourth of the length of a nucleosome sequence), similarly to results for yeast [3], [13]. The two patterns predicted 71% of nucleosomes but only 26% of nucleosomes were predicted by both patterns (Table 1). Two {AA, TT} patterns of yeast [4] did not have significant correlations with human nucleosome sequences (Supplementary Fig. S10b). By comparison, alternative nucleosome positioning code model (duration Hidden Markov model, HMM) proposed by [20], [21] predicted 41% of nucleosomes in the interval ±36 bp. 15% of sequences were predicted as a nucleosome free region (NFR) using this model. The maximum of predicted nucleosomes using HMM and human dinucleotide patterns match with the center of nucleosome sequences, 4 times more than with shuffled sequences, Z score>20 (Supplementary Fig. S10c).

**Table 1:** Portion of predicted nucleosomes by two {AA, TT} and {GG, CC} patterns of human nucleosome and HMM [21].

Precision	GG,CC pattern	AA,TT pattern	AA,TT or GG,CC patterns	AA,TT and GG,CC patterns
±36 bp	0.4768	0.5041	0.7163	0.2646
	HMM	AA,TT pattern	HMM or AA,TT pattern	HMM and AA,TT pattern
±36 bp	0.4137	0.5028	0.6955	0.221
	HMM	GG, CC pattern	HMM or GG, CC pattern	HMM and GG, CC pattern
±36 bp	0.4137	0.4772	0.6819	0.209

### 7. Correlation of dinucleotide patterns and promoter sequences

We separated experimental nucleosome sequences 146 bp long of normal CD4^+^ and apoptotic T-cells obtained by [8] and [14] from 32,038 promoter regions 2000 bp long (±1000 bp from TSS). Each set of sequences was divided into two subsets according to normal (one nucleosome per 200 nucleotides, 16670 TSS) or low (one or less than one nucleosome per 1000 nucleotides, 15368 TSS) nucleosome occupancy in promoters. We calculated the correlation of {AA, TT, AT} and {GG, CC, GC} dinucleotide patterns with two groups of promoters with normal and low nucleosome occupancy (Supplementary Fig. S11). Distribution of correlation between {AA, TT} patterns and promoter sequences in the two kinds of promoters with normal or low nucleosome occupancy were similar. The distribution had two peaks: upstream and downstream from TSS. The position of the downstream peak was 50 bp from TSS. It was located 40 bp upstream from the position of +1 nucleosomes received experimentally from normal CD4^+^ cells. The position of the upstream peak was 50 bp from TSS and matches to the site known as nucleosome free region of human promoters. Distribution of correlation between {GG, CC} patterns and promoter sequence differed in the two kinds of promoters with normal or low nucleosome occupancy. Distribution of correlation between {GG, CC} pattern obtained from normal cells had two peaks similar to the {AA, TT} pattern. With that the position of the downstream peak was closer to the position of +1 nucleosome. Distribution of correlation between {GG, CC} pattern and DNA obtained from apoptotic and non-apoptotic promoter sequences had only one peak 50 bp upstream from TSS and did not have peaks related to +1 nucleosome. We observed correlation between dinucleotide patterns and DNA sequence at the position of the nucleosome (Supplementary Fig. S11). However, distribution of correlation differed from the actual positions of the nucleosomes around TSS: a peak that matched with a nucleosome free region and a shift of 40 bp between downstream peak and position of +1 nucleosome (Supplementary Fig. S11). Therefore we assume that other transcription elements factors such as transcription factor binding proteins, RNA polymerase II and chromatin remodeling complex are related to displacement and/or removal of nucleosomes from preferable positions around TSS.

### 8. Structure of promoters of apoptotic genes during apoptosis

**Figure 4:**
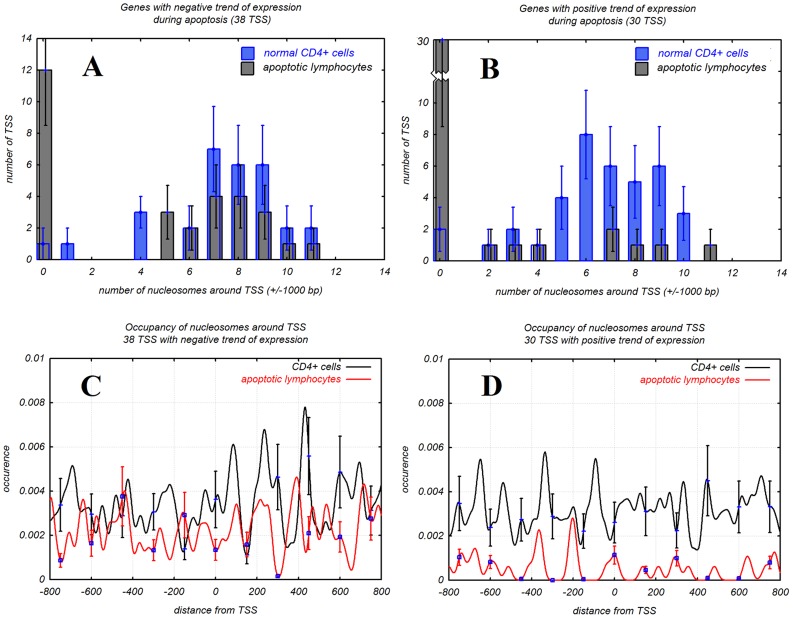
Number of nucleosomes on promoters of differentially regulated apoptosis genes: (A) – genes with low expression, (B) - genes with high expression during apoptosis. Distribution of nucleosomes around TSS of genes with positive (C) and negative (D) trend of expression during apoptosis.

We examined 68 promoters of 52 genes related to apoptosis obtained from [22]. The genes have positive or negative changes of expression during apoptosis (Supplementary Table S1). Promoter sequences were obtained from DBTSS (see methods) and data on expression of the genes was obtained from [22]. We found that promoters of genes with a positive trend of expression had much less nucleosomes during apoptosis than they had in CD4^+^ cells under normal conditions (Fig. 4b). At the same time, promoters of genes with a negative trend of expression during apoptosis had a similar number of nucleosomes to those in CD4^+^ cells in normal conditions (Fig. 4a). Average numbers of nucleosomes in promoters with a negative trend of expression were 7.3 and 4.5 in normal CD4^+^ cells and apoptotic lymphocytes, respectively. These promoters lost one third of nucleosomes during apoptosis. Average numbers of nucleosomes in promoters with a positive trend of expression were 6.5 and 1.5 in normal CD4^+^ and apoptotic lymphocytes, respectively. The promoters of highly expressed genes lost much more nucleosomes (77%) during apoptosis than promoters of low expressed genes (Table 2). These promoters lost almost all nucleosomes on the downstream region from TSS (Fig. 4d). Promoters of genes with a negative trend of expression mostly retained nucleosomes during apoptosis and had synchronized nucleosomes downstream from TSS (Fig. 4c).

**Table 2:** Nucleosome occurrence per promoter (±1000 bp around TSS).

	Average number of nucleosomes per promoter	Number of nucleosomes	Number of promoters
Normal T cell (up regulated genes)	6.5	246	38
Apoptotic T cell (up regulated genes)	1.3	51	38
Normal T cell (down regulated genes)	7.3	218	30
Apoptotic T cell (down regulated genes)	4.5	135	30

### 9. AA-TT and GG-CC dinucleotide patterns and intrinsic DNA curvature

Analysis of intrinsic DNA curvature of nucleosomal sequences revealed that DNA sequences of nucleosomes from normal cells predicted by {AA, TT} pattern have sites that are more curved at ±55 and ±65 bases from dyad symmetry (Supplementary Fig. S12). We used 11 bp window to calculate DNA curvature. DNA sequences of nucleosomes from apoptotic cells predicted by {AA, TT} pattern are more curved. We suggest that the curved sites at the end of nucleosome sequences stabilize nucleosomes during apoptosis. DNA sequences of +1 nucleosomes predicted by {AA, TT} pattern have higher gradient of curvature than the entire nucleosome set. DNA sequences of nucleosomes predicted by {GG, CC} pattern have different distribution of curvature. They have a less sharp gradient of curvature but a higher curvature site at ±15 bases from the dyad symmetry. Sites ±55 and ±65 bases from the dyad match with kinked DNA in nucleosome as observed in X-ray experiments [23] and theoretically [24] whereas sites ±15 bases from the dyad match with predicted kinked DNA in theoretical analysis only [24].

## Discussion

### 1. Comparison of two experimental sets of human nucleosomes

Analysis of apoptotic and normal T-cells revealed that almost 50% of promoters lost nucleosomes (Fig. 1 and 2) during apoptosis. The promoters that lost nucleosomes were GC-rich whereas promoters retaining nucleosomes were AT-rich (Supplementary Fig. S1). It was shown that GC-rich DNA is more favorable to formation of nucleosomes [10], [25]. GC-rich chromatin is more flexible [25] and GC-rich genes tend to be more active. GC-rich chromatin contains more modified histones such as acetylated histones H3 and H4 [26]. We observed that it has high level of disassembling during apoptosis. Process of disassembling GC-rich chromatin is fast and starts before apoptotic nuclease digests significant amounts of chromatin. The yield of digesting GC-rich chromatin is 20% of the AT-rich one. Not only GC-rich promoters but all GC-rich DNA sequence lost most of its nucleosomes during apoptosis (Supplementary Fig. S3). The first explanation is that nucleosome-free DNA is easier to degrade. But that does not explain why AT-rich DNA saved most nucleosomes when GC-rich DNA lost them. We observed that when genes related to apoptosis have tendency for high expression during apoptosis they lost nucleosomes while when they have tendency for low expression they kept nucleosomes. One of the possibilities why gene expression increases when promoters lose nucleosomes is because they have less negative regulation factors (the nucleosomes). On the other hand plasticity of expression of these genes is low. It is possible that high expression of apoptosis related genes is more important than plasticity during apoptosis because of it being the final process of a dying cell. Other parameters of regulation do not matter; the goal is to finish the process as quickly as possible.

### 2. Dinucleotide pattern of +1 nucleosome

It's well known that +1 nucleosome is better phased than other nucleosomes [8], [27]. Not only DNA pattern determines position of +1 nucleosome but other regulatory elements as RNA polymerase or chromatin remodelling complex [8], [18]). We observed a shift of +1 nucleosome from preferable position determined by nucleosomal patterns. Patterns obtained from +1 nucleosomes were periodical with a period of about 10 bp as in canonical nucleosome patterns. We suggest that the periodicity of 10 bp of the 200 bp long region downstream of TSS [28] is essential for shifting of +1 nucleosomes and remodelling chromatin around TSS. Periodical TSS downstream region is longer than a nucleosome sequence. Nucleosomes are not shifted randomly but shifted by multiples of 10 nucleotides as 10, 20, 30 bp etc. from the pattern-preferable position. RNA polymerase evicts nucleosomes from NFR and shifts +1 nucleosomes downstream from TSS by tens of bases during transcription [18]. This observation is consistent with the lack of nucleosomes upstream from TSS (NFR) and in a short interval downstream from TSS where we observe high correlation between nucleosome patterns and sequences (Supplementary Fig. S11). New position of +1 nucleosome is determined by other non-canonical patterns. {AA, TT} pattern of +1 nucleosome has an unusual peak at dyad symmetry. This central peak could be explained by the fact that +1 nucleosomes are moved along DNA sequence with periodical AA-TT dinucleotides. In this case AA-TT dinucleotides can be relocated to dyad symmetry which is not observed in canonical AA-TT pattern. All AA and TT dinucleotides are located in places where the DNA minor groove faces the histone octamer. Six of these positions 16/17, 26/27, 38/39, 109/110, 121/122, and 131/132 are identified as kinked [23]. Two other positions 57/58 and 88/89 were predicted as kinked too [24]. The minor groove in the two last positions is extremely narrow. On the other hand it is known that 10 bp periodical AA-TT dinucleotides have the largest DNA curvature [29]. The bent sites of DNA are delocalized throughout the nucleosome core region and have varying degrees of intrinsic curvature [30]. Intrinsic curvature of DNA around kinked sites assists forming and stabilizing nucleosome structure. {GG, CC} pattern of +1 nucleosome from AT-rich promoters have different changes. CC dinucleotide follows GG dinucleotide instead of their nonadjacent alternating arrangement in canonical pattern. GG-CC dinucleotides are flexible [25] and located between the deformed kinked sites of DNA sequence of +1 nucleosome (Fig. 3) unlike conventional nucleosomes where they alternate every 5 nucleotides (Fig. 2). Analysis of distribution of three and tetra nucleotides revealed that AAT, ATT, TTA, TAA, AATT, and TTAA have the same position as AA and TT dinucleotides. GGC, GCC and GGCC have the same position as GG and CC dinucleotides, other CCG, CGG, and CCGG oligomers were not found.

### 3. Correlation between dinucleotide patterns and experimental positions of nucleosomes in promoters

Human nucleosomes have a periodical pattern for each of the 16 dinucleotides. Combinations of {AA, TT} and {GG, CC} are the patterns that have the best correlation with nucleosomal sequences. Both of these patterns have two peaks of maximum correlation between dinucleotide patterns and DNA sequences in human promoters. The peaks are positioned 50 bp upstream and 50 bp downstream from TSS. Upstream peak matches with nucleosome free region (NFR), and the downstream peak relates to +1 nucleosome. Despite of the upstream region having maximum correlation with {AA, TT} and {GG, CC} nucleosome patterns it has a NFR. Apparently not only DNA sequence determines nucleosome position but other transcription regulatory factors as well. Transcription binding proteins and RNA polymerase displace nucleosomes from NFR and shift +1 nucleosomes by tens of bases downstream from preferable position [8]. Both {AA, TT} patterns obtained from normal and apoptotic cells have the same position of peaks (maximum correlation) around TSS while {GG, CC} patterns have different distribution of correlation with promoter sequences. Distribution of correlation between {GG, CC} pattern obtained from normal T-cells and promoter sequences has similar two peaks around TSS as {AA, TT} patterns. Downstream peak is slightly closer to the position obtained by experiment. {GG, CC} pattern obtained from apoptotic lymphocytes had only one peak at the upstream region and did not have a downstream peak. This means that the apoptotic cells do not have +1 nucleosomes in GC-rich promoters.

### 4. Conclusion

Apoptotic lymphocytes lost 80% of nucleosomes in GC-rich promoters (50% of all promoters) and GC-rich DNA compared with normal CD4^+^ cells. The rate of disassembling chromatin of GC-rich promoters and all GC-rich DNA was high; thus they lost these nucleosomes before defragmentation of DNA by apoptotic nuclease takes place. For this reason, nucleosomes from apoptotic cells had higher occurrence of AA and TT dinucleotides than from normal CD4^+^ cells. Apoptotic related genes such as caspase cascade or P53 signaling pathway were highly expressed and their promoters lost 80% of nucleosomes.

We compared nucleosome occupancy around TSS in apoptotic lymphocytes and normal CD4+ cells obtained by [8], [13], [ and 14], and we could see similarities and differences between these sets. GC-rich promoters lost nucleosomes during apoptosis like highly expressed genes in [13]. These two groups of promoters have the same genes (Supplementary Fig. S2). AT-rich promoters in [13] have three times higher nucleosome occupancy than GC-rich, (Z-score = 10, Pe<0.001). With that nucleosome occupancy in two groups of promoters obtained by [14]) do not have so significant difference (Figure 1). All three sets have clear visible maxima of +1 nucleosomes at the distance 100–120 bp downstream from TSS. We suggest that genes with GC-rich promoter have higher expression than AT-rich in normal and apoptotic conditions.

Both sets of nucleosomal sequences possessed similar dinucleotide distributions.

All dinucleotide distributions: AA, TT, GG, CC and WW, SS (W = A, T and S = G, C) and RR, YY (R is Purine, Y is Pyrimidine), had periodicity ∼10 bp (Supplementary Fig. S4 and S5). +1 nucleosomes from normal T-cells had alternating phase of GG and CC dinucleotides whereas +1 nucleosomes from apoptotic lymphocytes had adjacent GG-CC peaks. {AA, TT} pattern of +1 nucleosomes from normal CD4^+^ cells had a central peak of AA and TT dinucleotides matching with dyad symmetry.

## Methods

### 1. Nucleosome preparation

We analyzed two sets of human nucleosome DNA fragments. The first set was an isolated mononucleosome-sized DNA from MNase-digested chromatin. This set was sequenced using Solexa sequencing technology by [8]. Unique non paired nucleosomal sequences 24–25 bp long were obtained as described previously [31] from the normal CD4^+^ T lymphocytes. The raw data consists of roughly 40 million non-paired reads 24–25 bases long. Data was obtained from the Short Read Archive (SRA, Illumina sequencing of Human resting genomic fragment library, NCBI) [http://www.ncbi.nlm.nih.gov/sra/SRX000168], under accession number SRA000234.

The second set was chromatin DNA of peripheral blood lymphocytes (from a human male) cleaved by apoptotic nucleases and fractionated by electrophoresis on an agarose gel [14]. Bands of ∼200 bp length consisting of mononucleosomal DNA were excised. These fragments were ligated to GAII paired end adaptors by the standard Illumina protocol, amplified by PCR and sequenced on a 2nd Gen Illumina GAII sequencer. The raw data consisted of roughly 44 million paired reads of 120 bases long.

### 2. Position of nucleosomal sequence on genome

Positions of raw data sequences 24–25 bases long [8] and 120 bases long [14] were matched to the *H. sapiens* genome by BLAT [http://genome.ucsc.edu/cgi-bin/hgBlat?command=start].

### 3. Human genome

The genome sequence of male *H. sapiens* (22 paired, X and Y chromosomes) of total length 2,865,822,365 bp. was obtained from NCBI genome archive [ftp://ftp.ncbi.nih.gov/genomes/H_sapiens], BUILD.37.2, 22 November 2010.

### 4. Sequences around transcription start sites (TSS)

32,038 human promoter sequences 600 bp long (500 bp upstream and 100 bp downstream) were obtained from DBTSS [http://dbtss.hgc.jp/].

### 5. Position of nucleosome sequences and TSSs

Positions of promoter sequences 600 bases long (promoter sequences, DBTSS) were matched on the *H. sapiens* genome by BLAT [http://genome.ucsc.edu/cgi-bin/hgBlat?command=start]. Then positions of TSS were aligned with experimental nucleosome positions from [8], [13], [ and 14] on referral human genome. The sequences with 100% identity were used in further analyses.

### 6. Dinucleotide distribution on nucleosome sequences

Nucleosome sequences 147 bp long were cut from referral human genome starting from 5′ end of experimental position and aligned. Then occurrence of all 16 dinucleotides was calculated for each position.

### 7. Dinucleotide shuffling

The dinucleotide shuffling for each of 16 dinucleotide pairs was performed in order to estimate statistical significance of the results. The swap algorithm [32] was used to conserve dinucleotide composition because the used models of DNA property as bendability, intrinsic curvature, and sequence periodicity of dinucleotides depend on the dinucleotide composition of DNA.

### 8. Bootstrap estimation method

The calculations of the confidence interval using the classical estimation methods are not applicable if the estimator does not have an approximately normal distribution or the type of distribution is unknown. Some computational methods were developed for this case. One of useful methods is bootstrapping [33].

### 9. Prediction of nucleosome positions

Correlation of the dinucleotide patterns to particular DNA sequences was calculated according to the protocol described in [15]. Nucleosome positions were predicted as a maximum correlation between DNA sequence and dinucleotide nucleosomal patterns.

### 10. DNA curvature

DNA curvature was calculated as described by [29]. We used window 11 bp long to calculate the DNA curvature.

## Supporting Information

Figure S1 (**A**) Distribution of AA, TT, GG, CC occurrence and nucleosome's occupancy around 16670 TSS (first group of promoters). Gray shaded line represents nucleosome occurrence around TSS in CD4+ cells using [8], black line represents nucleosome occurrence in apoptotic lymphocytes using [14]. (**B**) Distribution of AA, TT, GG, CC occurrence and nucleosomes around 15368 GC-rich (second group) promoters. Gray shaded line represents nucleosome occurrence around TSS in CD4+ cells using [8], black line represents nucleosome occurrence in apoptotic lymphocytes using [14]. Left Y axe corresponds to dinucleotide occurrence, right Y axe corresponds to nucleosome occupancy.
(TIF)

Figure S2 (**A**) Distribution of nucleosomes around transcription start sites; black line corresponds to all TSS, blue one to AT-rich (group 1) and red one to GC-rich (group 2) promoters. Data are from [13].
(TIF)

Figure S3 Average distance between experimental positions of nucleosomes in normal and apoptotic T-cells on referral human genome. Coverage DNA by nucleosomes is 5 time less in GC-rich than in neutral or AT-rich sequence.
(TIF)

Figure S4 Fourier transform of AA-TT, GG-CC, WW (A, T) and SS (G, C) dinucleotide distributions. Apoptotic lymphocytes, data is from [14]; normal CD4^+^ cells, data is from [8].
(TIF)

Figure S5 Dinucleotide distributions of RR (Purine) and YY (Pyrimidine) dinucleotides around nucleosome dyad symmetry (left panel) have period ∼10 bp (Fourier transform, right panels), whole sets. Black line corresponds to apoptotic lymphocytes, data is from [14]; red line corresponds to CD4^+^ cells, data is from [8], gray line is dinucleotide shuffled sequence for comparison.
(TIF)

Figure S6 Correlation between (**A**) AA-TT, AA-AT, AA-TA; (**B**) GG-CC, GG-Gc, GG-CG; (**C**) WW-SS (A, T - G, C) and YY-RR (purine-pyrimidine) dinucleotide patterns of nucleosomes from normal CD4^+^ cells [8]. Each pattern was moved along paired pattern and correlation between them was used as measure on the distance from −73 to 73 bp (1/2 of length of the nucleosome pattern 146 bp long). Pairs of dinucleotides GG-CC, WW-SS and YY-RR alternate each 5 bp.
(TIF)

Figure S7 Distribution of length of DNA fragments obtained from [14]. Average length is 170 bp.
(TIF)

Figure S8 Distribution of WW (A, T) dinucleotides along DNA fragments with different length.
(TIF)

Figure S9 Prediction of position of nucleosomes obtained from apoptotic (bottom panels) and normal CD4^+^ cells (top panels) by {AA-TT-TA} (red line) and {GG-CC-GC} (black line) patterns, apoptotic lymphocytes, data is from [14]; CD4^+^ cells, data is from [8]. Blue line corresponds to shuffled sequence.
(TIF)

Figure S10 Prediction of position of nucleosomes: (**A**) by two AA, TT and GG, CC patterns of human nucleosomes obtained from normal CD4^+^ cells [8]; (**B**) two sub-patterns with positive and negative correlation with AA, TT patterns obtained from yeast by [4]; (**C**) HMM proposed by [20], [21]. Blue line corresponds to shuffled sequence.
(TIF)

Figure S11 Correlation of {AA, TT, AT} (left panels) and {GG, CC, GC} (right panels) dinucleotide patterns and two groups of promoters with normal (top panels) and low (bottom panels) occurrence of nucleosomes around promoter.
(TIF)

Figure S12 Distribution of DNA curvature around dyad symmetry of all and +1 nucleosomes from normal CD4^+^ T-cells and apoptotic lymphocytes. Red line corresponds to all nucleosomes of the set, blue one corresponds to nucleosomes predicted by AA-TT pattern, and black one corresponds to nucleosomes predicted by GG-CC pattern. Curvature window is 11 bp long.
(TIF)

Table S1 Expression trends of 52 differentially regulated apoptosis genes from [22].
(DOCX)
