# Supplementary figures and images for: Apoptotic Lymphocytes of H. sapiens Lose Nucleosomes in GC-Rich Promoters
Source: PLoS Comput Biol. 2014 Jul 31;10(7):e1003760. doi: 10.1371/journal.pcbi.1003760 (PMC4117428; doi:10.1371/journal.pcbi.1003760)

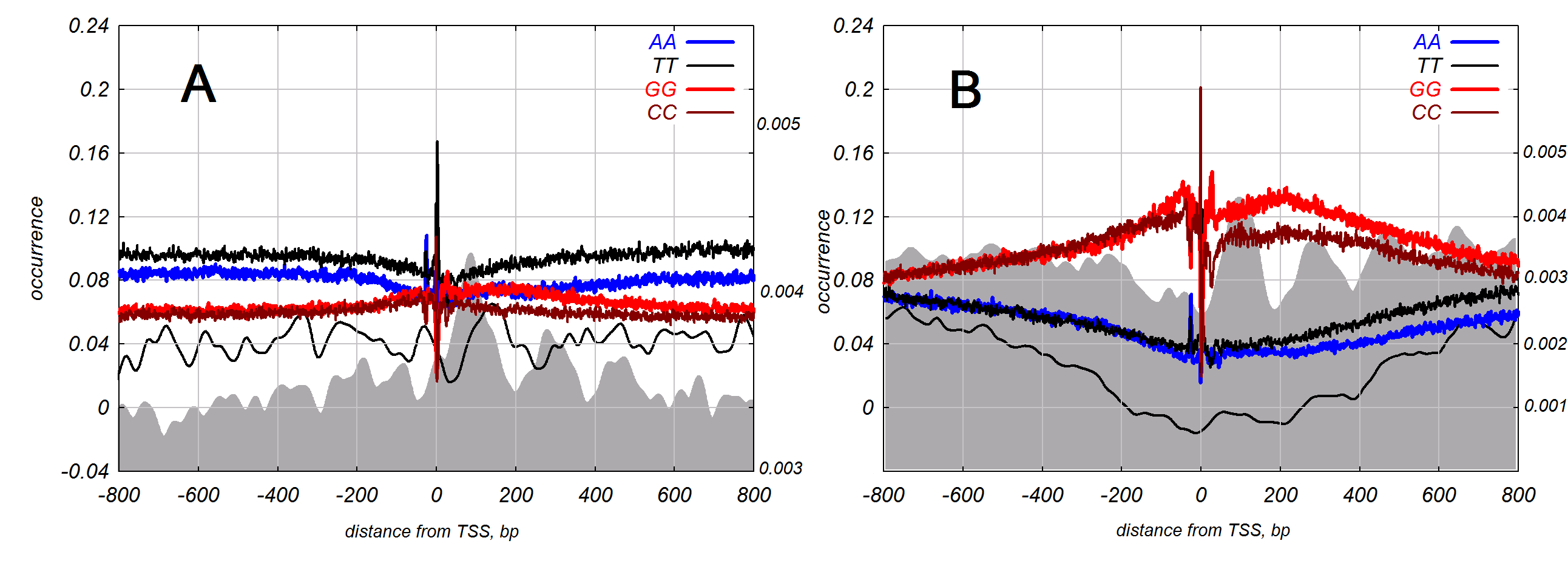

Supplement: Figure S1 — (A) Distribution of AA, TT, GG, CC occurrence and nucleosome's occupancy around 16670 TSS (first group of promoters). Gray shaded line represents nucleosome occurrence around TSS in CD4+ cells using [8], black line represents nucleosome occurrence in apoptotic lymphocytes using [14]. (B) Distribution of AA, TT, GG, CC occurrence and nucleosomes around 15368 GC-rich (second group) promoters. Gray shaded line represents nucleosome occurrence around TSS in CD4+ cells using [8], black line represents nucleosome occurrence in apoptotic lymphocytes using [14]. Left Y axe corresponds to dinucleotide occurrence, right Y axe corresponds to nucleosome occupancy. (TIF) [file pcbi.1003760.s001.tif]

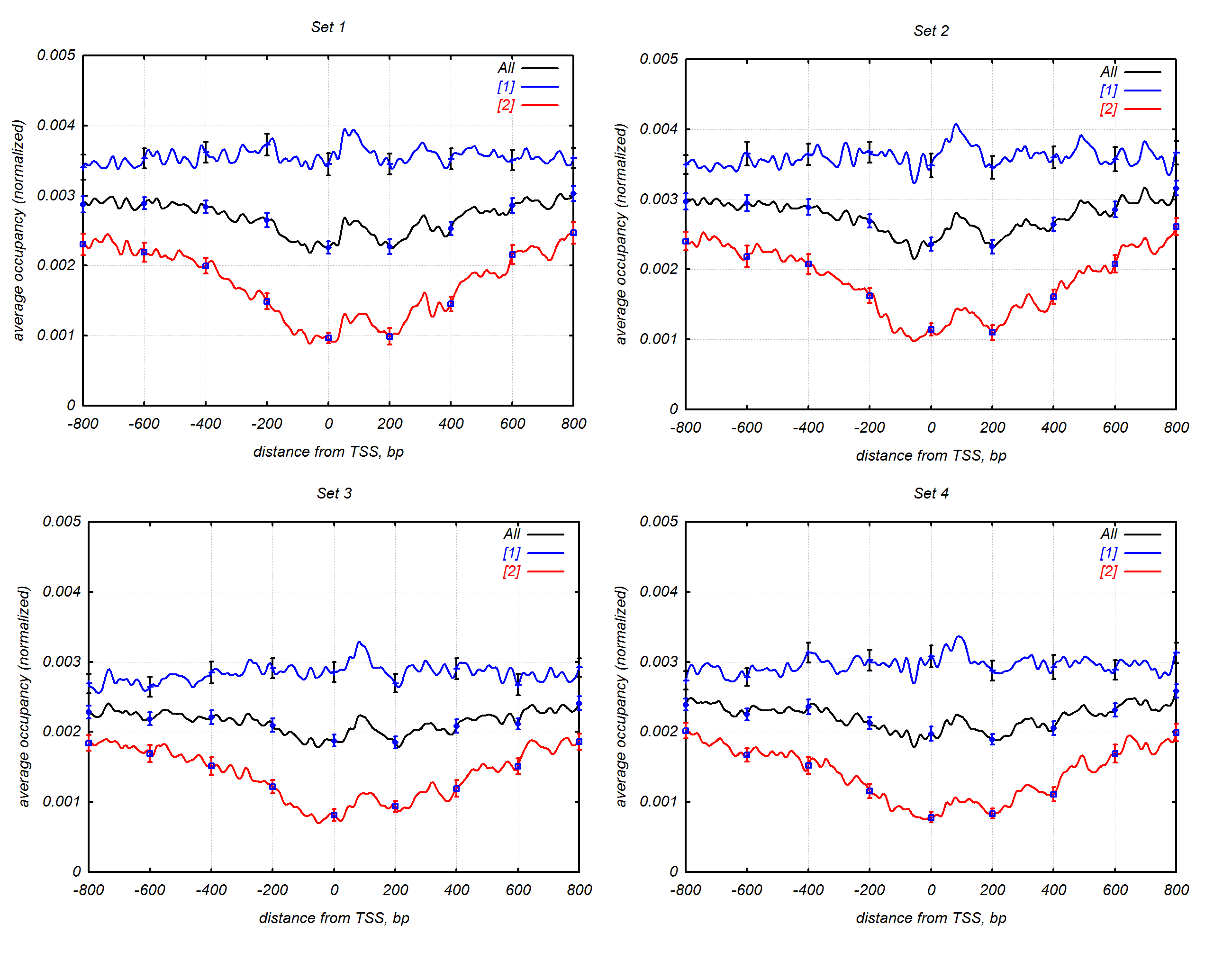

Supplement: Figure S2 — (A) Distribution of nucleosomes around transcription start sites; black line corresponds to all TSS, blue one to AT-rich (group 1) and red one to GC-rich (group 2) promoters. Data are from [13]. (TIF) [file pcbi.1003760.s002.tif]

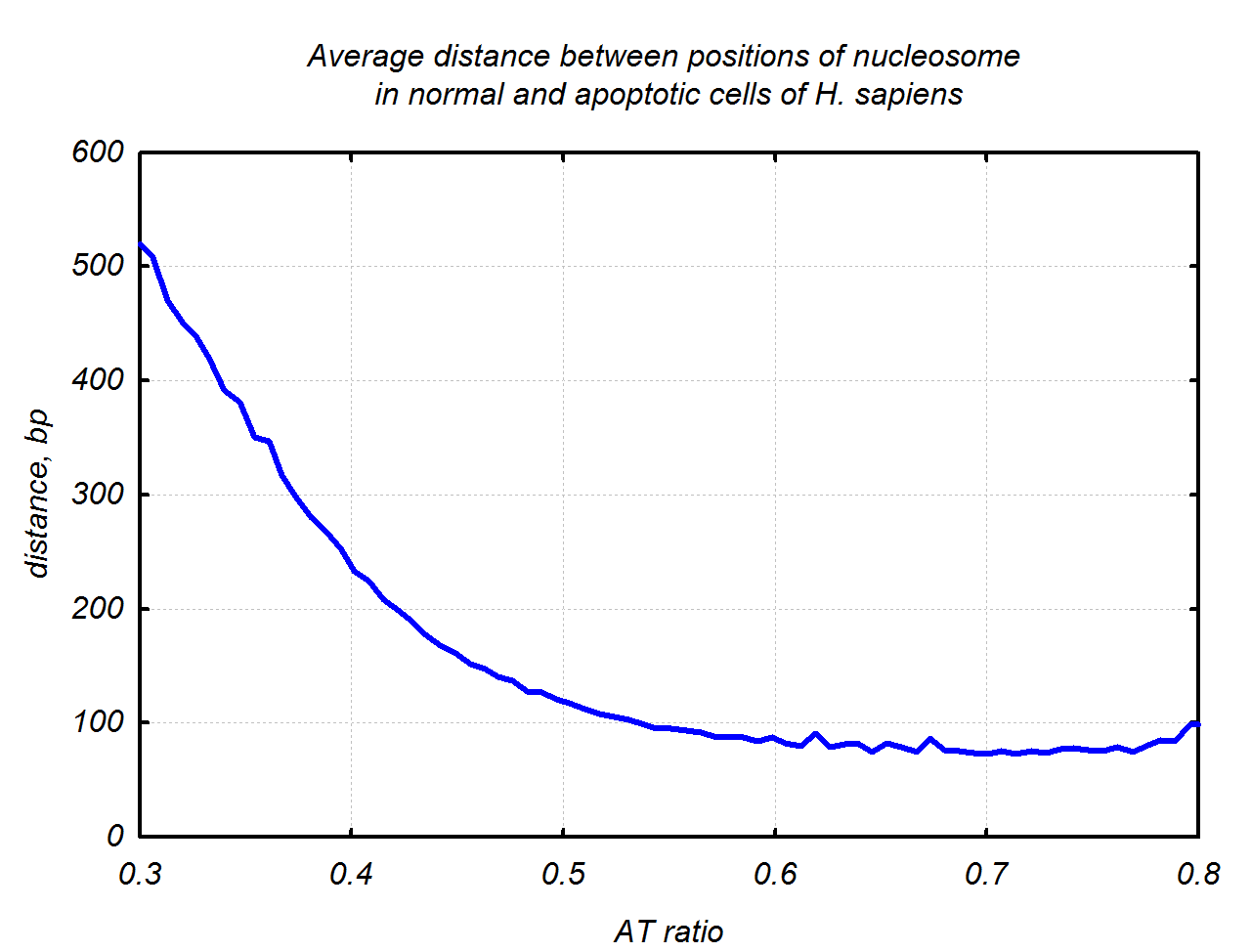

Supplement: Figure S3 — Average distance between experimental positions of nucleosomes in normal and apoptotic T-cells on referral human genome. Coverage DNA by nucleosomes is 5 time less in GC-rich than in neutral or AT-rich sequence. (TIF) [file pcbi.1003760.s003.tif]

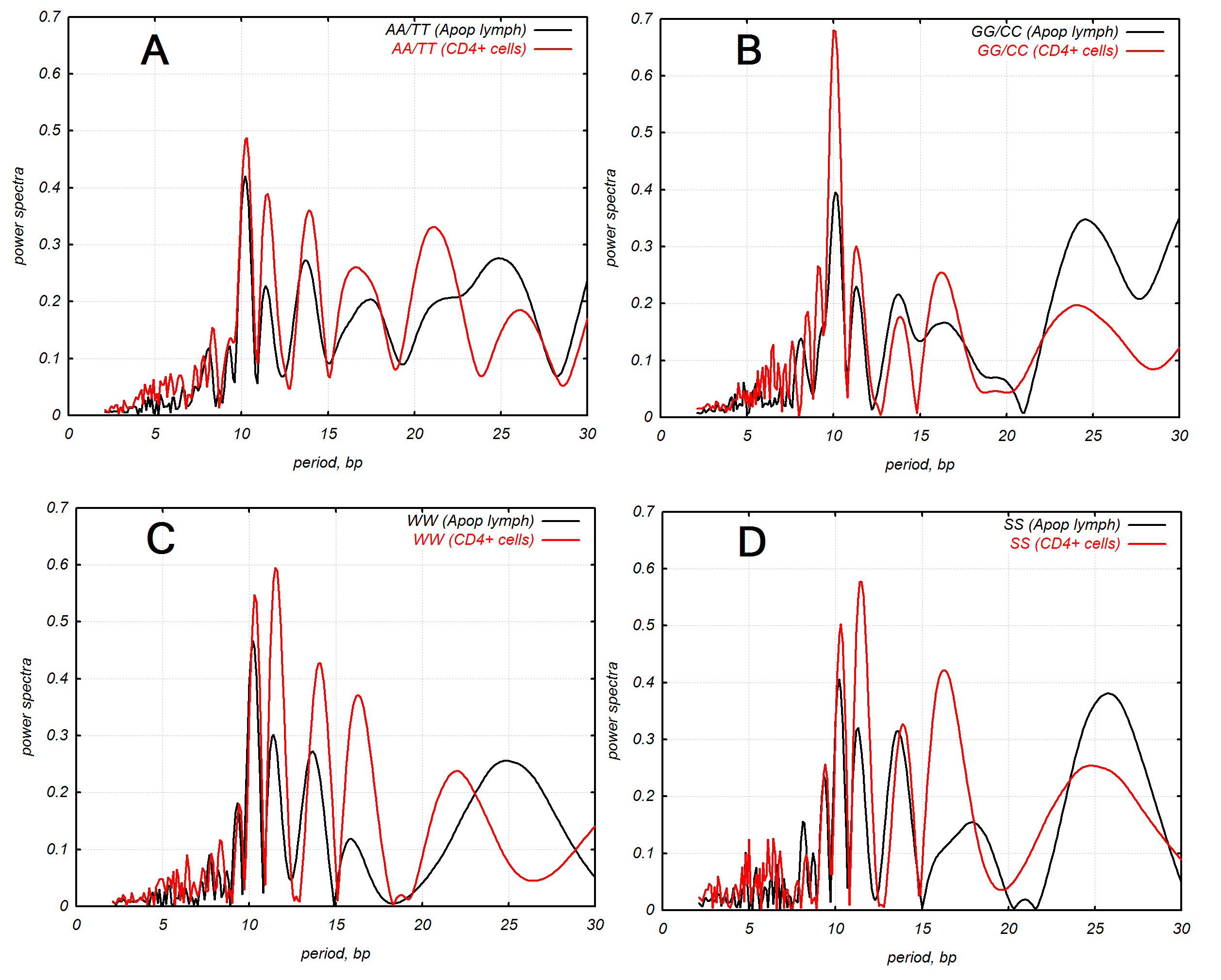

Supplement: Figure S4 — Fourier transform of AA-TT, GG-CC, WW (A, T) and SS (G, C) dinucleotide distributions. Apoptotic lymphocytes, data is from [14]; normal CD4+ cells, data is from [8]. (TIF) [file pcbi.1003760.s004.tif]

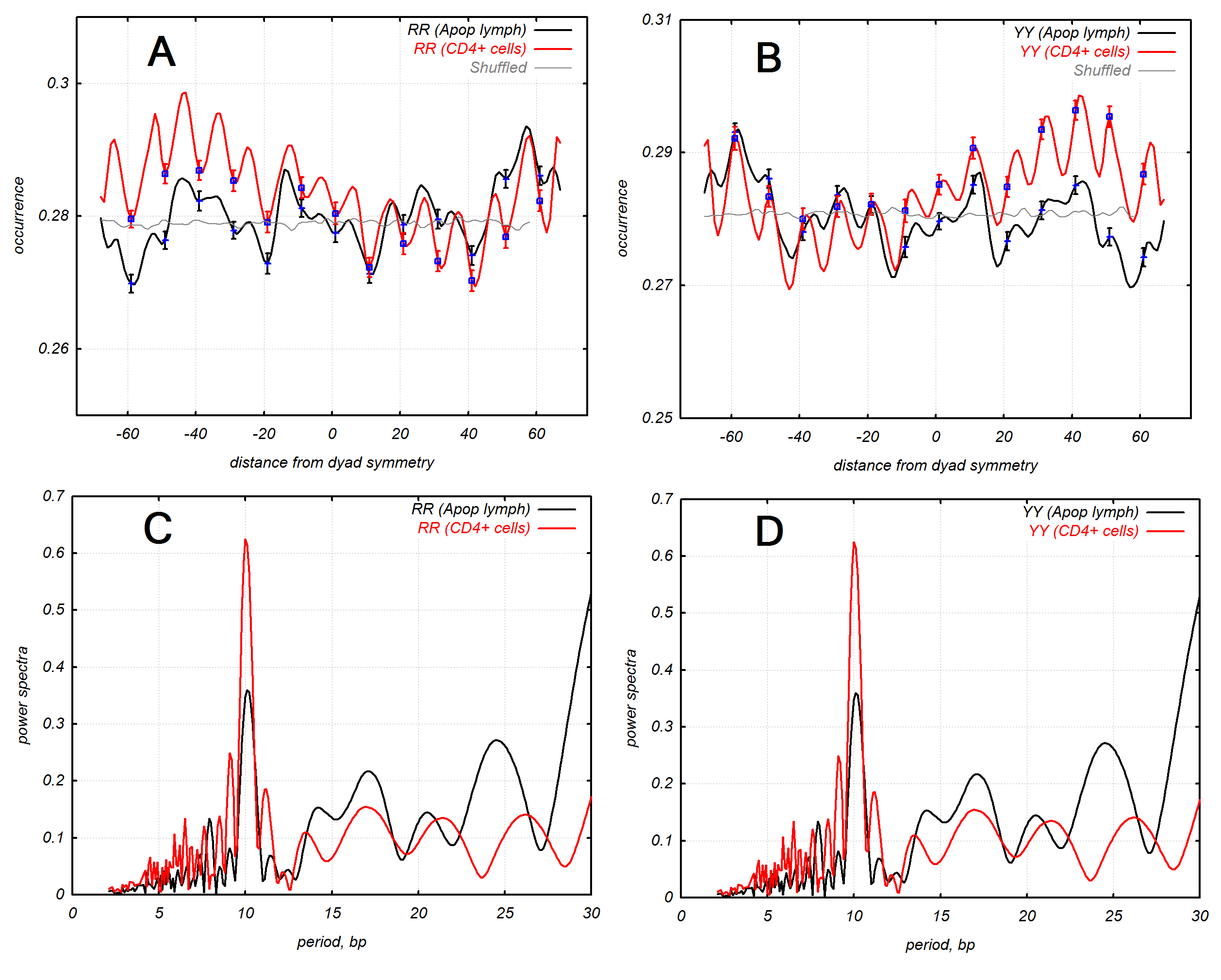

Supplement: Figure S5 — Dinucleotide distributions of RR (Purine) and YY (Pyrimidine) dinucleotides around nucleosome dyad symmetry (left panel) have period ∼10 bp (Fourier transform, right panels), whole sets. Black line corresponds to apoptotic lymphocytes, data is from [14]; red line corresponds to CD4+ cells, data is from [8], gray line is dinucleotide shuffled sequence for comparison. (TIF) [file pcbi.1003760.s005.tif]

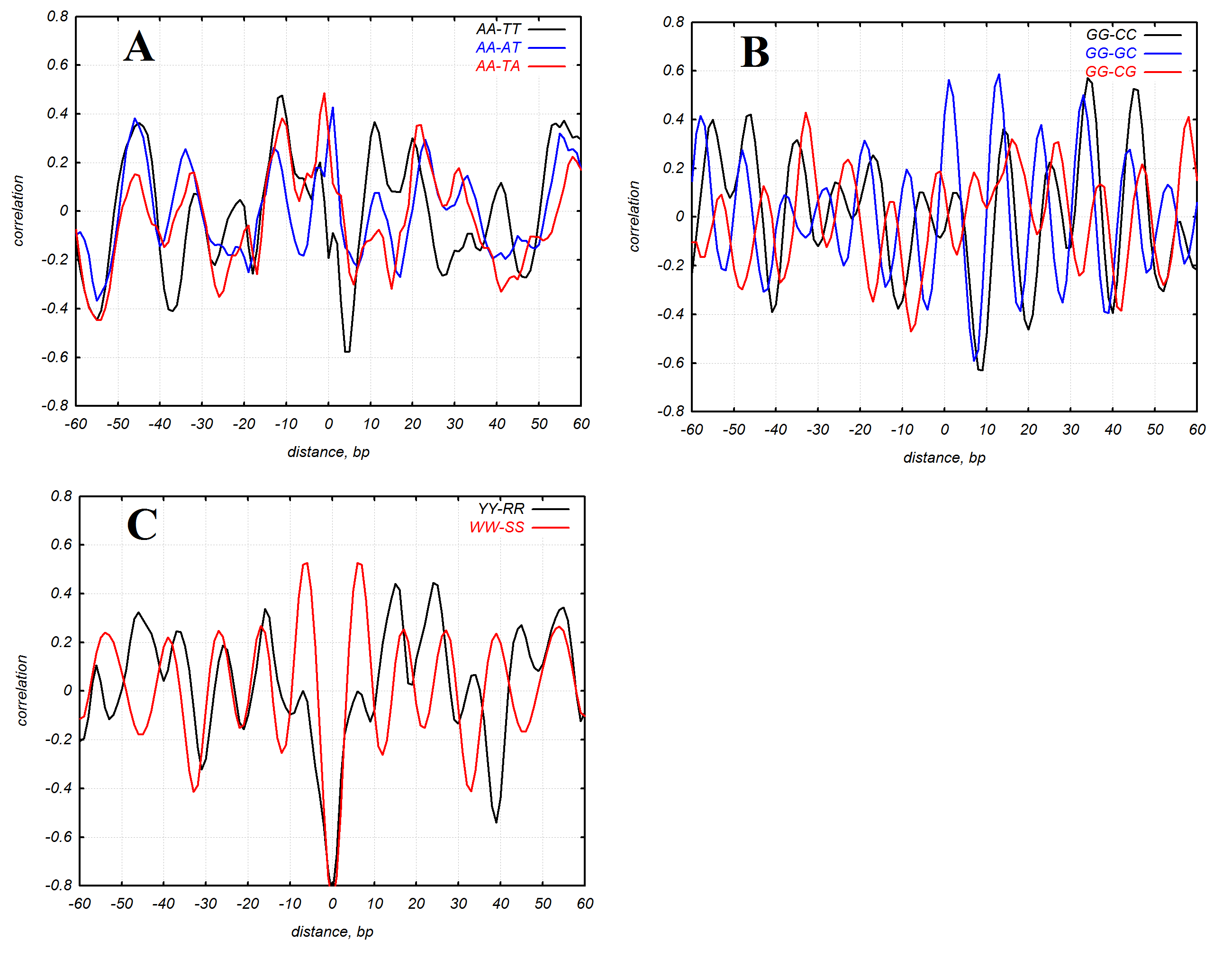

Supplement: Figure S6 — Correlation between (A) AA-TT, AA-AT, AA-TA; (B) GG-CC, GG-Gc, GG-CG; (C) WW-SS (A, T - G, C) and YY-RR (purine-pyrimidine) dinucleotide patterns of nucleosomes from normal CD4+ cells [8]. Each pattern was moved along paired pattern and correlation between them was used as measure on the distance from −73 to 73 bp (1/2 of length of the nucleosome pattern 146 bp long). Pairs of dinucleotides GG-CC, WW-SS and YY-RR alternate each 5 bp. (TIF) [file pcbi.1003760.s006.tif]

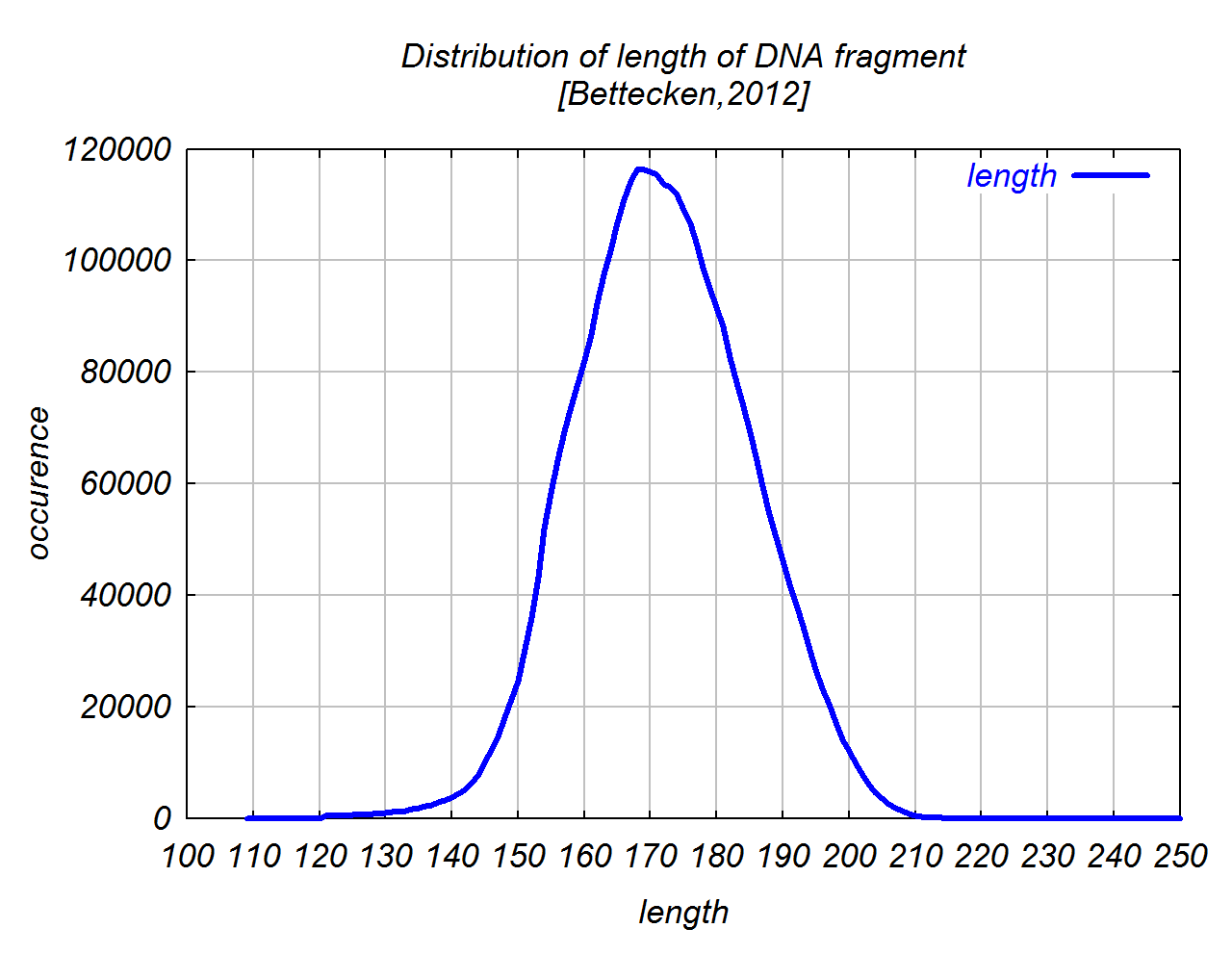

Supplement: Figure S7 — Distribution of length of DNA fragments obtained from [14]. Average length is 170 bp. (TIF) [file pcbi.1003760.s007.tif]

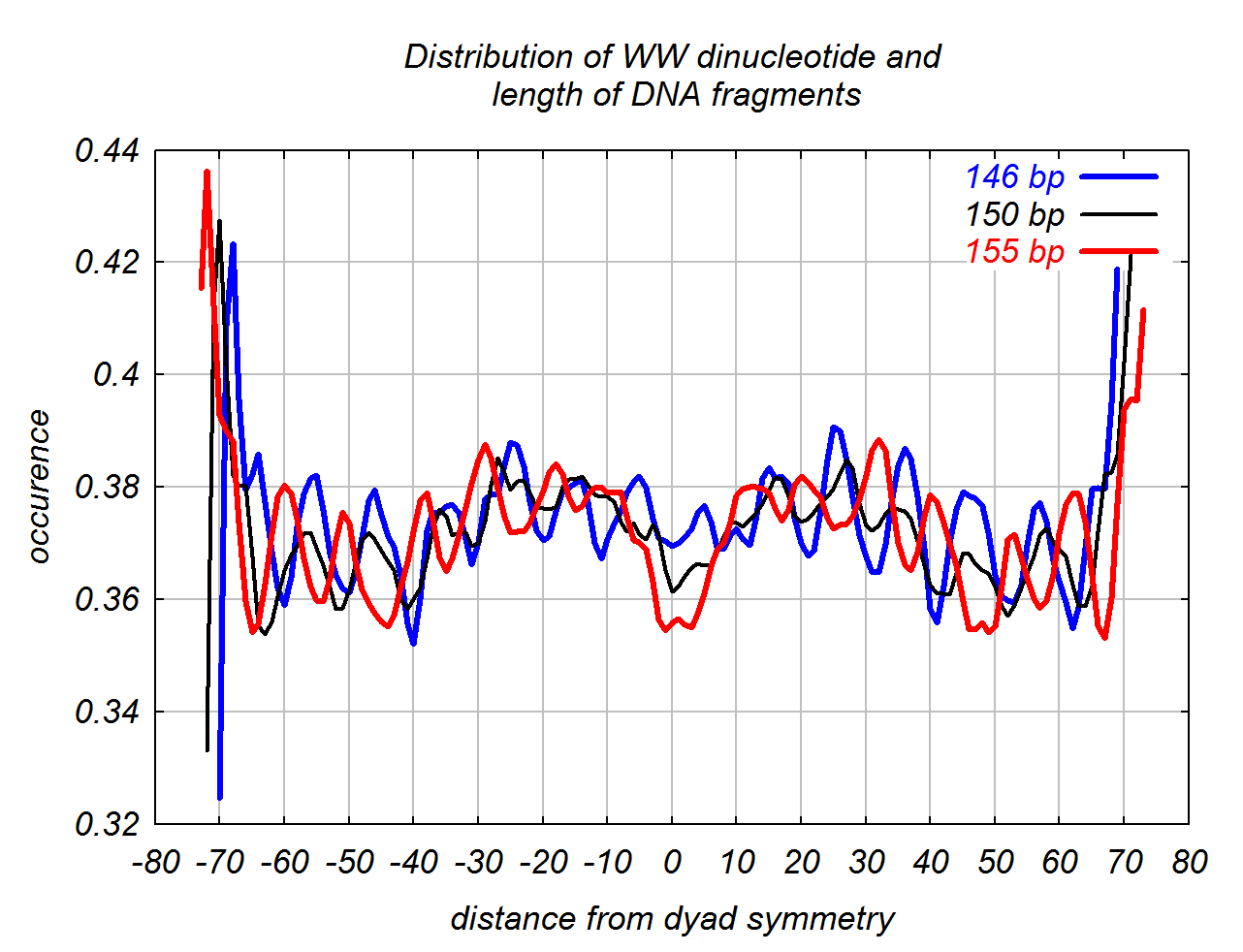

Supplement: Figure S8 — Distribution of WW (A, T) dinucleotides along DNA fragments with different length. (TIF) [file pcbi.1003760.s008.tif]

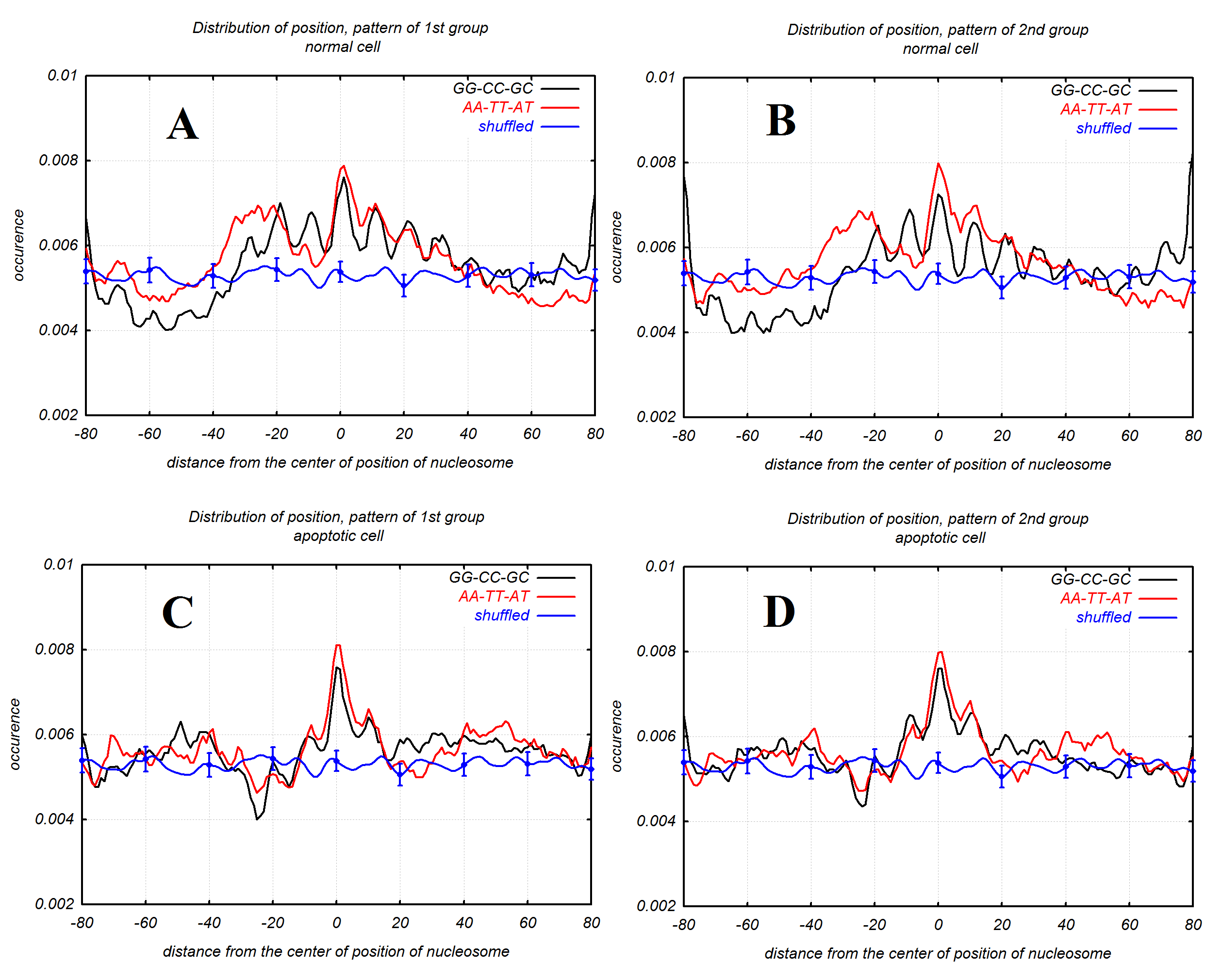

Supplement: Figure S9 — Prediction of position of nucleosomes obtained from apoptotic (bottom panels) and normal CD4+ cells (top panels) by {AA-TT-TA} (red line) and {GG-CC-GC} (black line) patterns, apoptotic lymphocytes, data is from [14]; CD4+ cells, data is from [8]. Blue line corresponds to shuffled sequence. (TIF) [file pcbi.1003760.s009.tif]

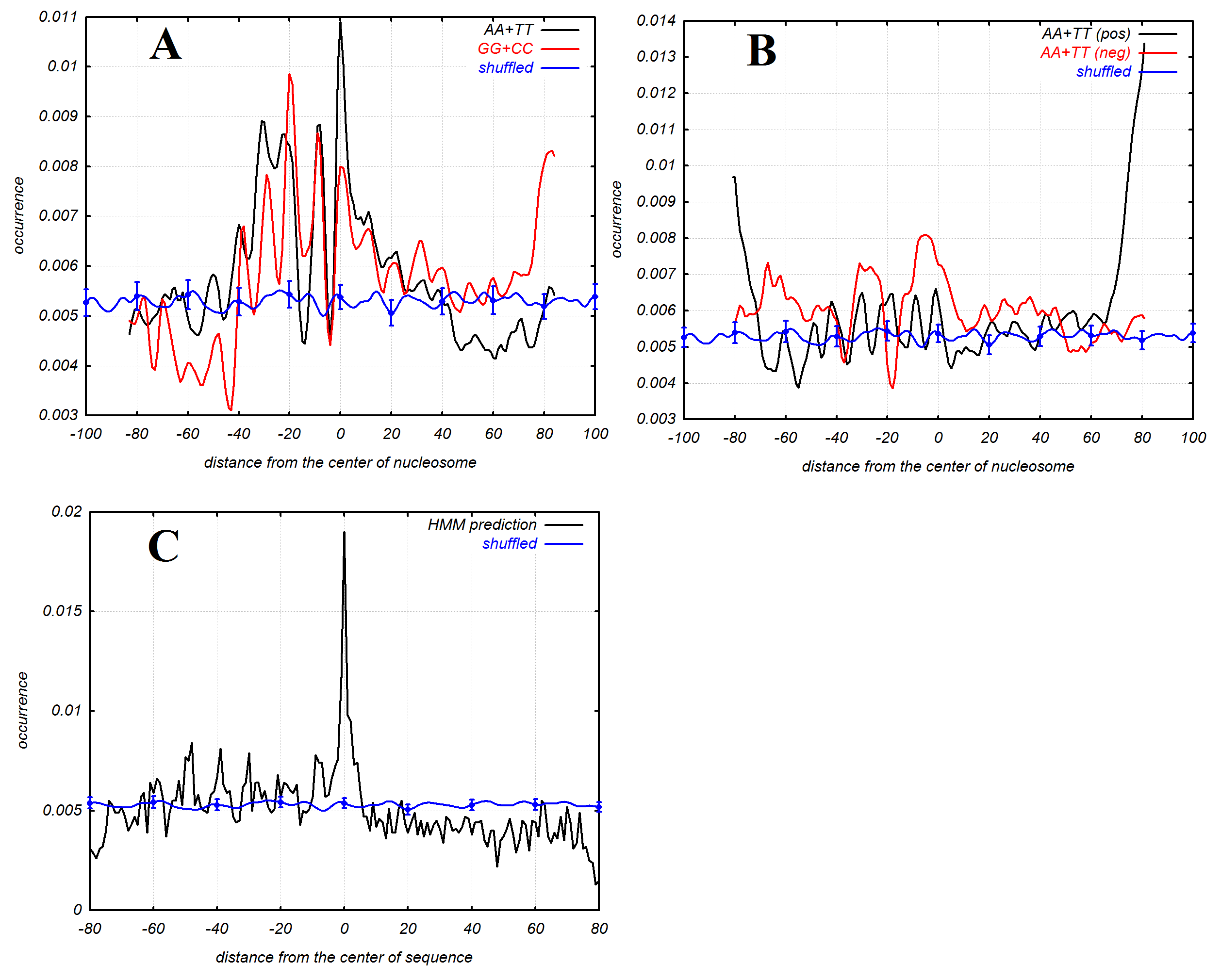

Supplement: Figure S10 — Prediction of position of nucleosomes: (A) by two AA, TT and GG, CC patterns of human nucleosomes obtained from normal CD4+ cells [8]; (B) two sub-patterns with positive and negative correlation with AA, TT patterns obtained from yeast by [4]; (C) HMM proposed by [20], [21]. Blue line corresponds to shuffled sequence. (TIF) [file pcbi.1003760.s010.tif]

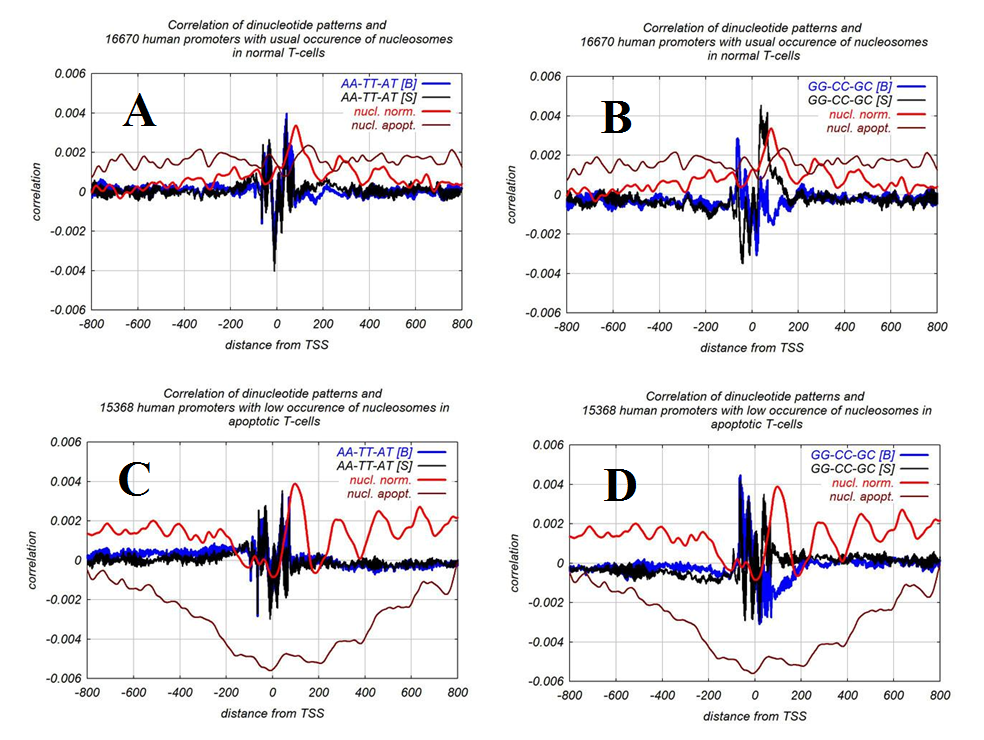

Supplement: Figure S11 — Correlation of {AA, TT, AT} (left panels) and {GG, CC, GC} (right panels) dinucleotide patterns and two groups of promoters with normal (top panels) and low (bottom panels) occurrence of nucleosomes around promoter. (TIF) [file pcbi.1003760.s011.tif]

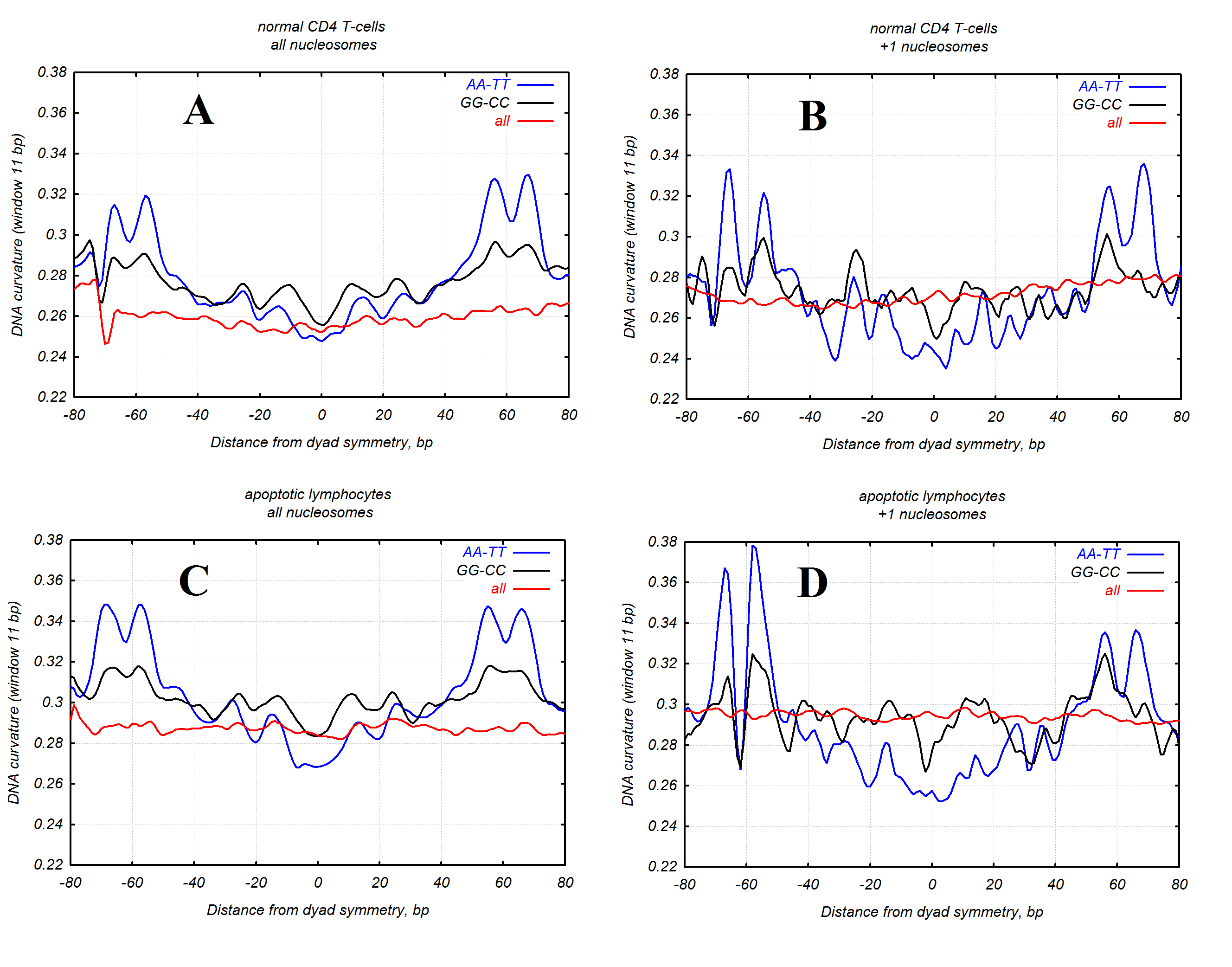

Supplement: Figure S12 — Distribution of DNA curvature around dyad symmetry of all and +1 nucleosomes from normal CD4+ T-cells and apoptotic lymphocytes. Red line corresponds to all nucleosomes of the set, blue one corresponds to nucleosomes predicted by AA-TT pattern, and black one corresponds to nucleosomes predicted by GG-CC pattern. Curvature window is 11 bp long. (TIF) [file pcbi.1003760.s012.tif]
