# Supplementary material for: Apoptotic Lymphocytes of H. sapiens Lose Nucleosomes in GC-Rich Promoters
Source: PLoS Comput Biol. 2014 Jul 31;10(7):e1003760. doi: 10.1371/journal.pcbi.1003760 (PMC4117428; doi:10.1371/journal.pcbi.1003760)
Supplement: Table S1 — Expression trends of 52 differentially regulated apoptosis genes from [22]. (DOCX) [file pcbi.1003760.s013.docx]

**Table S1.** Expression trends of 52 differentially regulated apoptosis genes (from Jailwala at al. 2009).

| name of gene | type | Change of expression  TD1/control (log) | Z score |
| --- | --- | --- | --- |
| CDKN1B | AKT-FOXO3A pathway | 1 | 105 |
| CITED2 | AKT-FOXO3A pathway | 1 | 33 |
| STK4 | AKT-FOXO3A pathway | 1.1 | 33 |
| FOX01A | AKT-FOXO3A pathway | 1.2 | 66 |
| FOX03A | AKT-FOXO3A pathway | 1.3 | 44 |
| SESN1 | AKT-FOXO3A pathway | 1.3 | 55 |
| INFRSF10B | AKT-FOXO3A pathway | 2.2 | 68 |
| CCND2 | AKT-FOXO3A pathway | -1.6 | -34 |
| AKT | AKT-FOXO3A pathway | -2.2 | -37 |
| BIM | P53 signaling pathway | 1 | 42 |
| DYRK2 | P53 signaling pathway | 1.1 | 103 |
| CADD45A | P53 signaling pathway | 1.3 | 37 |
| CADD45B | P53 signaling pathway | 1 | 71 |
| STK17A | P53 signaling pathway | 1.1 | 32 |
| STK17B | P53 signaling pathway | 1.3 | 48 |
| SESN3 | P53 signaling pathway | 1.5 | 56 |
| BCL2 | Activation of caspase cascade | -1.5 | -21 |
| CYCs | Activation of caspase cascade | 1.1 | 51 |
| BCL10 | Activation of caspase cascade | 1.6 | 46 |
| FAS | Activation of caspase cascade | 1.4 | 29 |
| STCH | stress responsive genes | 2.2 | 67 |
| SOD2 | stress responsive genes | 1.2 | 109 |
| SERP1 | stress responsive genes | 1.2 | 50 |
| HSPA9 | stress responsive genes | 1.3 | 48 |
| HSPA14 | stress responsive genes | 1.3 | 129 |
| HSP90 | stress responsive genes | 1 | 182 |
| HSF2 | stress responsive genes | 1.5 | 80 |
| HLA-DRA | HLA genes | -2.1 | -22 |
| HLA-DRB1 | HLA genes | -1.5 | -53 |
| HLA-DQB1 | HLA genes | -1.6 | -33 |
| HLA-E | HLA genes | -1.1 | -33 |
| HLA-A | HLA genes | -1.1 | -23 |
| GIAMP8 | GIAMP gene family | -1.7 | -41 |
| GIAMP7 | GIAMP gene family | -1.5 | -21 |
| GIAMP6 | GIAMP gene family | -1.7 | -71 |
| GIAMP5 | GIAMP gene family | 1.7 | -54 |
| GIAMP4 | GIAMP gene family | -2.6 | -77 |
| GIAMP1 | GIAMP gene family | -2.4 | -37 |
| IL10RB | Cytokine and chemokine receptors | -1.6 | -36 |
| IL18R1 | Cytokine and chemokine receptors | -1.4 | -34 |
| IL1R1 | Cytokine and chemokine receptors | -2.2 | -26 |
| IL27RA | Cytokine and chemokine receptors | -1.5 | -26 |
| IL2RA | Cytokine and chemokine receptors | -2.1 | -24 |
| IL2RB | Cytokine and chemokine receptors | -1.2 | -38 |
| IL2RG | Cytokine and chemokine receptors | -1.5 | -29 |
| IL6R | Cytokine and chemokine receptors | -2.3 | -40 |
| ILR7 | Cytokine and chemokine receptors | -1.4 | -37 |
| CCR2 | Cytokine and chemokine receptors | -4 | -68 |
| CCR5 | Cytokine and chemokine receptors | -2.9 | -75 |
| CCR6 | Cytokine and chemokine receptors | -1.7 | -35 |
| CXCR3 | Cytokine and chemokine receptors | -2.3 | -31 |
| CXCR6 | Cytokine and chemokine receptors | -2.1 | 32 |
